# Automated CMR Quantification of Pulmonary Artery Size-Based and Slow-Flow Metrics in Pulmonary Hypertension

**DOI:** 10.3390/diagnostics16193257

**Published:** 2026-10-08

**Authors:** Khalid Alghamdi, Alireza Hokmabadi, Rob van der Geest, Pankaj Garg, Turki Alnasser, Daniel J. Taylor, Michael Sharkey, Smitha Rajaram, David G. Kiely, Samer Alabed, Andrew J. Swift

**Affiliations:** 1 School of Medicine & Population Health, The University of Sheffield, Sheffield S10 2TN, UK; a.hokmabadi@sheffield.ac.uk (A.H.);; 2 Department of Radiological Sciences, College of Applied Medical Sciences, Imam Abdulrahman Bin Faisal University, Dammam 34212, Saudi Arabia; 3 Insigneo Institute, Faculty of Engineering, The University of Sheffield, Sheffield S10 2TN, UK; 4 Division of Image Processing, Department of Radiology, Leiden University Medical Centre, 2333 Leiden, The Netherlands; 5 Norwich Medical School, Faculty of Medicine and Health Sciences, University of East Anglia, Norwich NR4 7TJ, UK; 6 Radiological Sciences Department, College of Applied Medical Science, King Saud bin Abdulaziz University for Health Science, Riyadh 11426, Saudi Arabia; 7 King Abdullah International Medical Research Center (KAIMRC), Riyadh 11426, Saudi Arabia; 8 National Institute for Health and Care Research, Sheffield Biomedical Research Centre, Sheffield S10 2TN, UK; 9 Department of Clinical Radiology, Sheffield Teaching Hospitals NHS Foundation Trust, Sheffield S10 2TN, UK; 10 Sheffield Pulmonary Vascular Disease Unit, Sheffield Teaching Hospitals NHS Foundation Trust, Sheffield S10 2TN, UK

**Keywords:** pulmonary hypertension, cardiovascular magnetic resonance, pulmonary artery, slow-flow, automated segmentation, automated quantification, deep learning

## Abstract

**Background/Objectives:** Pulmonary artery (PA) dilatation and slow-flow are imaging features of pulmonary hypertension (PH), but their quantitative assessment on routine cardiovascular magnetic resonance (CMR) remains limited. This study aimed to develop and evaluate an automated CMR pipeline for quantifying PA size-based and slow-flow metrics from routine white-blood (WB) and black-blood (BB) acquisitions. **Methods:** A total of 205 CMR acquisitions (106 WB and 99 BB) were used for model development and independent testing. Separate deep learning nnU-Net-based segmentation models were developed for WB and BB acquisitions using a two-stage cascaded framework. Automated measurements included PA volume and PA slow-flow. The trained models were subsequently applied to a larger cohort of 1471 patients using available WB and BB acquisitions to assess automated measurement extraction and agreement with clinical reports. **Results:** Automated PA volumetric measurements demonstrated excellent agreement with manual reference measurements for both the WB and BB models (ICC 0.94 and 0.93, respectively), with high segmentation accuracy (Dice 0.86 and 0.80, respectively). Automated slow-flow measurements also demonstrated excellent volumetric agreement (ICC 0.94), despite lower spatial overlap (Dice 0.61). Automated slow-flow classification demonstrated 83% agreement with qualitative clinical reporting (κ = 0.58). **Conclusions:** Automated PA volumetric quantification from routine CMR is feasible. Automated slow-flow quantification showed promising performance but requires validation across scanner vendors and BB acquisition protocols before broader clinical application.

## 1. Introduction

Pulmonary hypertension (PH) is a progressive disease associated with changes in the pulmonary circulation, including pulmonary artery (PA) dilatation and altered pulmonary arterial blood flow [1]. Cardiovascular magnetic resonance (CMR) can capture these changes through quantification of PA morphology and pulmonary arterial haemodynamics [2,3].

PA dilatation is a recognised feature of PH and is incorporated into current diagnostic assessment, with the main PA diameter and pulmonary artery-to-aorta (PA/Ao) ratio used as established imaging markers of disease severity and prognosis [1]. Although PA diameter and the PA/Ao ratio are widely used, they are derived from a single imaging plane and may be influenced by slice selection, measurement location and reader variability [4]. In contrast, volumetric assessment captures the entire vessel and may provide a more comprehensive and reproducible measure of pulmonary vascular remodelling.

Among the flow-related features visible on CMR, PA slow-flow on black-blood (BB) imaging appears as persistent intraluminal high signal, reflecting sluggish pulmonary arterial blood flow. Previous studies have demonstrated its diagnostic and prognostic value in PH [5,6]. However, current assessment relies on visual interpretation or semi-quantitative scoring, which remain subjective and do not provide a standardised quantitative measure of slow-flow [7]. Automated segmentation could address this limitation by translating this qualitative imaging feature into a continuous volumetric measurement, enabling reproducible quantification of slow-flow within the PA.

Recent advances in automated CMR analysis have enabled reliable quantification of cardiac structure and function [8,9,10]. However, comparatively less attention has been given to automated pulmonary vascular assessment, with existing work focused on conventional phase-contrast flow quantification [11,12]. To our knowledge, no fully automated method has been developed to quantify PA slow-flow or to integrate it with PA size-based measurements from routine CMR.

Therefore, this study aimed to develop a fully automated CMR segmentation pipeline for quantifying PA slow-flow from BB CMR and PA volumetric and diameter-based measurements from white-blood (WB) CMR, and to evaluate its performance in an independent test dataset and a large retrospective clinical cohort.

## 2. Methods

### 2.1. Ethical Approval

The study was conducted in accordance with the Declaration of Helsinki and approved by the East of England–Cambridge East Research Ethics Committee (REC 22/EE/0011; 24 February 2022). The Assessing the Spectrum of Pulmonary Hypertension Identified at a Referral Centre (ASPIRE) registry is an ethically approved research database managed by Sheffield Teaching Hospitals NHS Foundation Trust (STH19747; REC 22/EE/0011). The study included UK Biobank data under Application Number 99895. Ethical approval for UK Biobank was granted by the North West Multi-centre Research Ethics Committee (REC 21/NW/0157). This study was reported in accordance with the Checklist for Artificial Intelligence in Medical Imaging (CLAIM) [13], and the completed checklist is provided as **Supplementary File S1**.

### 2.2. CMR Datasets and Acquisition 

**Figure 1:**
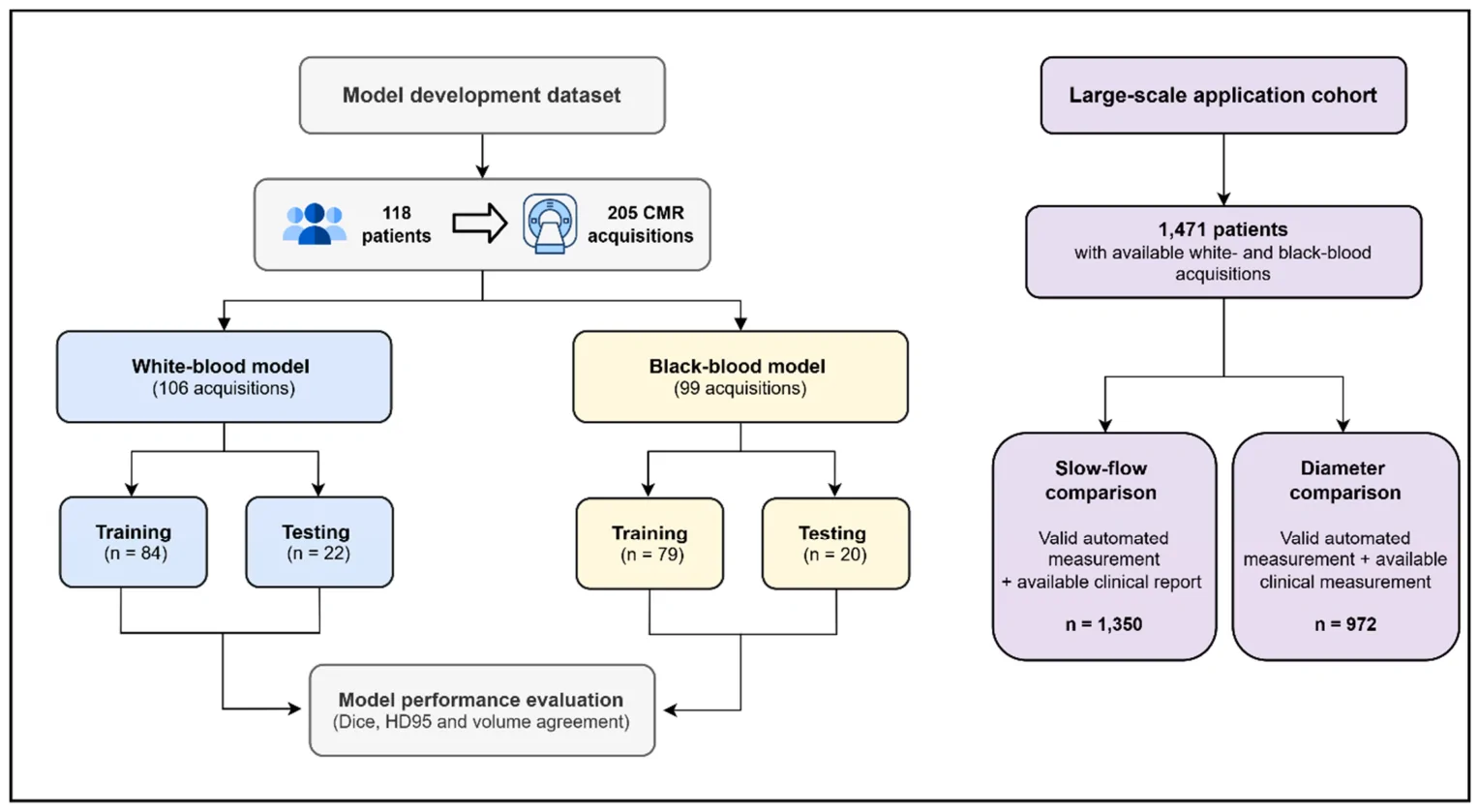
Overview of datasets used for model training, testing and deployment.

Two independent CMR datasets were used in this retrospective study, with no patient overlap between them. The first dataset comprised 205 CMR acquisitions, which were split into separate model development and independent test datasets. Dataset splitting was performed at the patient level, ensuring that no patient appeared in both the model development and independent test datasets. The second dataset comprised 1471 patients using available WB and BB acquisitions and was used for large-scale application of the trained models (**Figure 1**). Both datasets included scans acquired between 2008 and 2020 from patients with and without PH who underwent CMR as part of routine clinical assessment, as well as healthy volunteers who underwent CMR as part of a research protocol. All imaging data were de-identified prior to manual annotation and model development. Analyses were restricted to patients with the required CMR acquisitions and reference clinical measurements available for each comparison.

CMR examinations were acquired on 1.5T scanners from GE Healthcare and Siemens Healthineers using routine institutional protocols. On GE scanners, axial WB images were acquired using an ungated FIESTA sequence with TR/TE 3.2/1.4 ms, flip angle 55°, slice thickness 10 mm, no interslice gap, field of view 40 × 40 cm, matrix 200 × 200, and NEX 1.0. Axial BB images were typically acquired using an ECG-gated double inversion recovery fast spin-echo sequence with TR/TE 1332/50.6 ms, flip angle 90°, slice thickness 8 mm with 1 mm interslice gap, field of view 40 × 34 cm, matrix 160 × 144, and NEX 0.6. On Siemens scanners, WB images were acquired using a single-shot TrueFISP sequence, with a flip angle of 80°, slice thickness 8 mm, slice spacing 10 mm and matrix 256 × 154. Siemens BB acquisitions were not available for model development or testing.

### 2.3. Manual Segmentation

The PA, ascending aorta (AAo) and descending aorta (DAo) were manually segmented on WB and BB images, with an additional label assigned to the PA slow-flow region on BB images. Segmentations were performed across multiple axial slices, covering the major thoracic vessels to the cardiac valve plane.

Manual segmentation was performed by a trained radiographer (K.S., three years’ experience) and a senior radiologist (A.S., >10 years’ experience) using MASS software (Version 2025-EXP, Leiden University Medical Centre, Leiden, The Netherlands) with semi-automated tools, following a predefined segmentation approach. Where required, segmentations were reviewed and refined by the senior radiologist. These annotations were used as the reference standard for model training and evaluation.

Inter-observer repeatability was evaluated in a randomly selected subset of 30 acquisitions (15 WB and 15 BB), which were independently segmented by a second observer (S.R., >10 years’ experience) who was blinded to the original segmentations. Segmentation included the PA, AAo, and DAo on both image types, together with slow-flow regions on BB images. Spatial agreement between observers was assessed using Dice similarity coefficient and 95th percentile Hausdorff distance (HD95).

### 2.4. Model Development 

#### 2.4.1. Segmentation Model Development 

Separate segmentation models were developed for WB and BB CMR acquisitions. For each dataset, 80% of acquisitions were allocated to model development, while the remaining 20% were reserved for independent testing. WB acquisitions were used for PA and aortic volumetric quantification, with automated diameter measurements derived as part of an exploratory analysis, whereas BB acquisitions were used for PA volumetric and slow-flow quantification.

All images were reoriented to the right-anterior-superior (RAS) coordinate system to ensure consistent spatial orientation across acquisitions. WB images included annotations for the PA, AAo and DAo. The AAo and DAo were included as part of the aortic segmentation and centreline pipeline, with the AAo used to derive PA/Ao ratio measurements. BB images included the same vascular structures, together with an additional slow-flow label within the PA.

**Figure 2:**
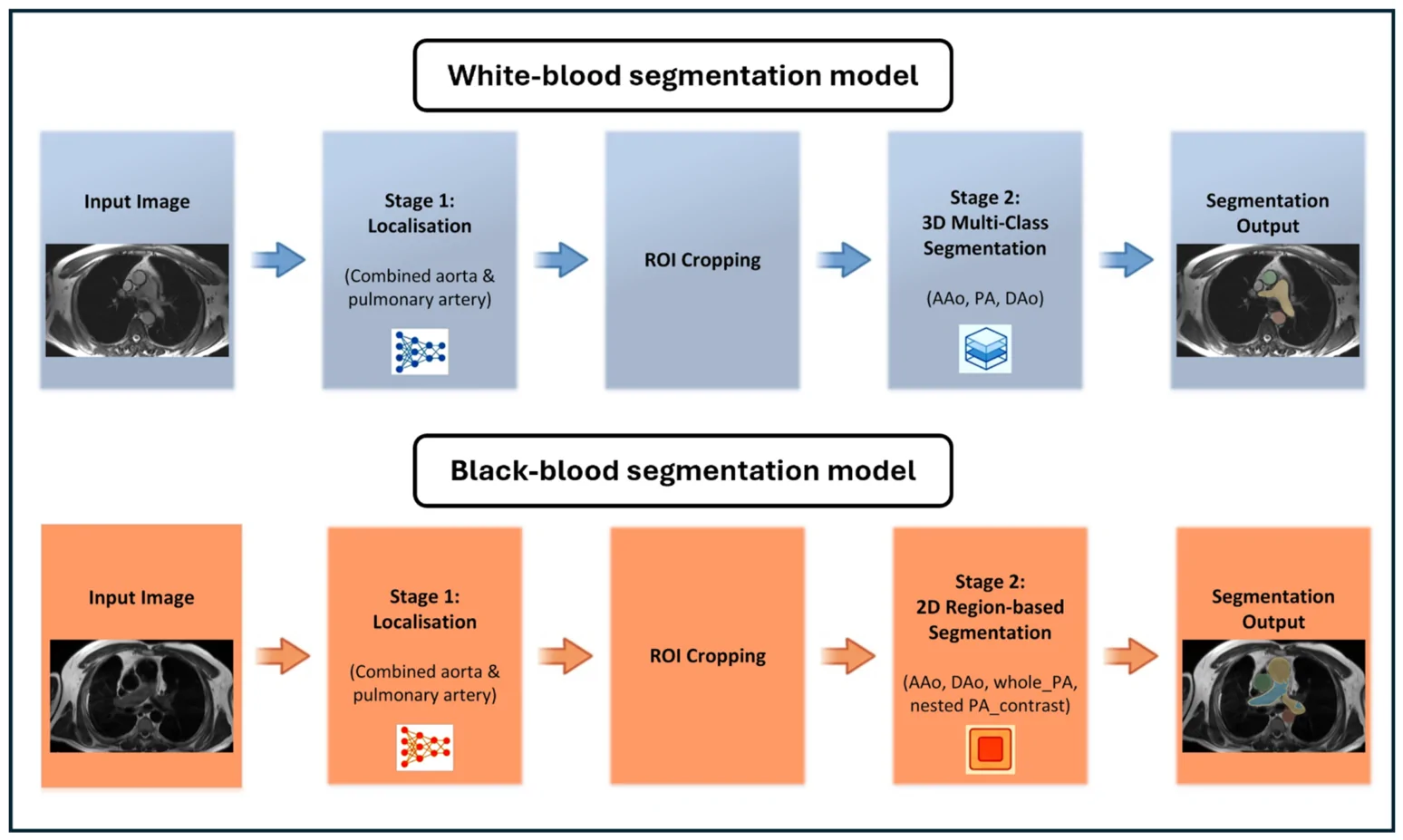
Two-stage cascaded segmentation pipeline for white-blood and black-blood CMR.

A two-stage cascaded segmentation pipeline based on the U-Net architecture was implemented [14]. The pipeline was implemented using the nnU-Net framework, with the cascaded design used to improve anatomical localisation and reduce computational complexity [15], as shown in **Figure 2**. In the first stage, a localisation model was trained to identify the combined PA and aortic region without distinguishing between individual vascular structures. The predicted region of interest was then used as boundaries to crop the input images and reduce background anatomy. These cropped outputs were subsequently used as inputs to the second-stage segmentation model, which performed detailed segmentation of the target vascular structures.

The second-stage model was trained using a standard multi-class segmentation approach to segment the PA, AAo and DAo on WB images. For the BB dataset, a region-based training strategy was employed, combining the PA with the slow-flow region under one label, with the slow-flow label treated as a nested subregion within the PA to account for their anatomical relationship.

All experiments were conducted on a Linux-based workstation equipped with NVIDIA H100 GPUs (96 GB memory), using Python 3.11, PyTorch 2.5.1, and CUDA 12.4. All models were trained from scratch without pretrained weights. Each localisation and segmentation model was trained for 250 epochs. First-stage localisation models were trained once for each acquisition type using the full training set. WB images were processed using a 3D full-resolution configuration with a batch size of three to exploit the available volumetric spatial information. In contrast, a 2D configuration with a batch size of 24 was adopted for BB acquisitions due to their limited through-plane coverage and relatively small number of slices. To improve model robustness and account for the relatively small training dataset, five-fold cross-validation was performed for the second-stage segmentation models, and final predictions were generated by ensembling the five trained models.

During training, standard nnU-Net data augmentation strategies, including random rotations, scaling, mirroring, elastic deformations, and Gaussian noise, were applied to improve model generalisability. Models were optimised using a hybrid loss function combining Dice similarity coefficient and cross-entropy terms. Foreground oversampling was used to mitigate class imbalance and improve the representation of smaller vascular structures and slow-flow regions during training.

Segmentation performance was evaluated by comparing automated segmentations with manual reference annotations. Spatial overlap was assessed using the Dice similarity coefficient, and boundary accuracy was assessed using the 95th percentile Hausdorff distance (HD95). To assess the anatomical localisation of automated slow-flow predictions, a containment analysis was performed in the independent BB test dataset. The whole-PA region was reconstructed as the union of the PA and slow-flow labels. The volume and proportion of predicted slow-flow outside the manual whole-PA region were calculated, including proportions >5 mm and >10 mm outside the PA, and false-positive volume was classified as within or outside the manual PA. To assess the contribution of extra-PA predictions to surface-distance error, HD95 was recalculated after restricting predictions to the manual whole-PA region. This analysis used manual reference information and was not considered a model performance metric. The trained models can be made available upon reasonable request to the corresponding author for research purposes by non-commercial entities.

#### 2.4.2. Volume-Based Measurement Extraction 

Volumetric measurements were extracted directly from the generated segmentation masks by summing all labelled voxels and multiplying by the corresponding voxel volume. This approach was used to derive PA and AAo volumes from the segmentation outputs, while PA slow-flow volume was extracted from the dedicated BB slow-flow label.

#### 2.4.3. Diameter-Based Measurement Extraction 

Diameter measurements were extracted from the WB CMR-derived vascular masks using a centreline-based geometric pipeline. The pipeline generated automated PA and AAo diameter measurements.

**Figure 3:**
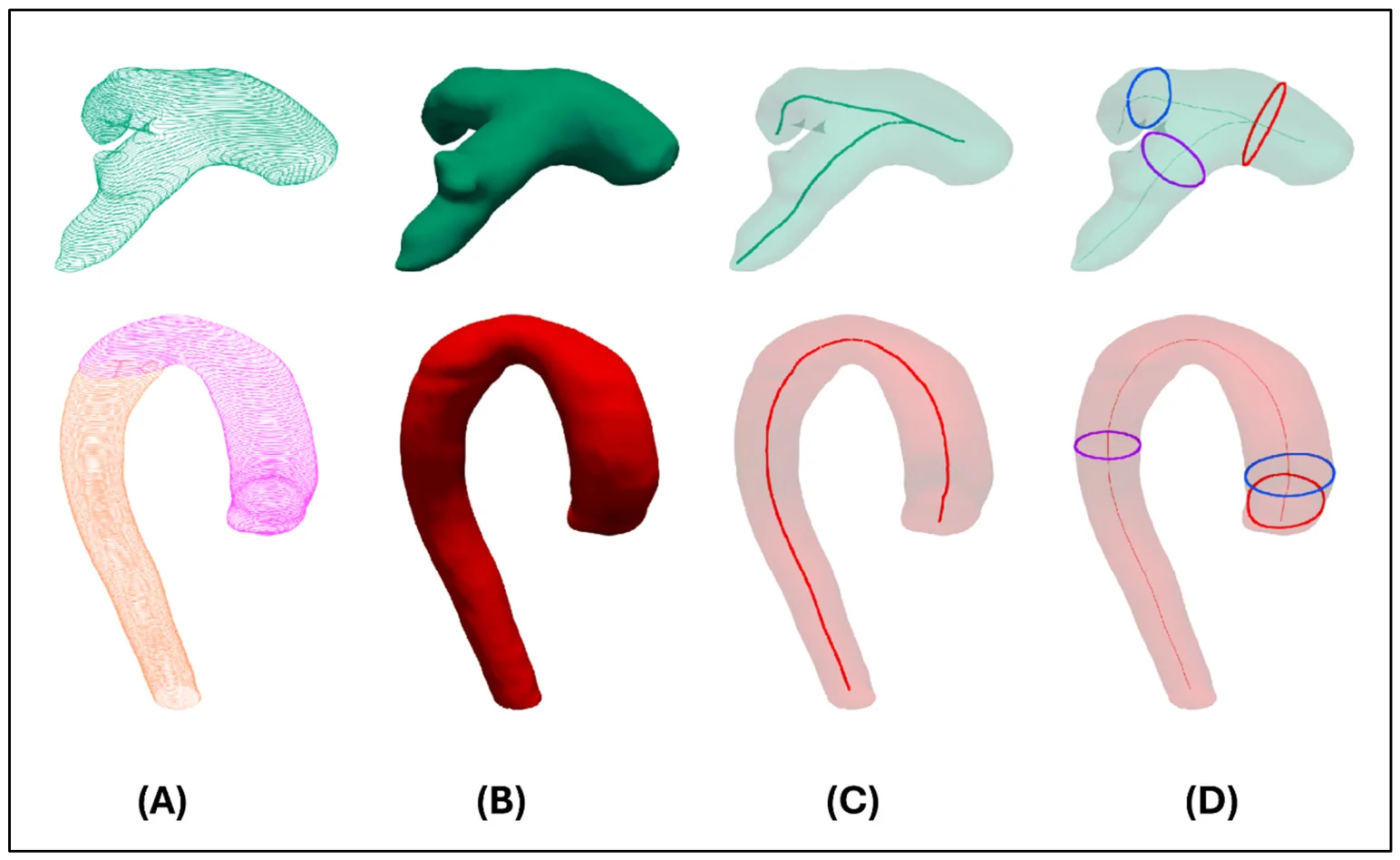
Diameter-based measurement pipeline for the pulmonary artery (top) and aorta (bottom). (**A**) Vessel contours from segmentation masks. (**B**) Three-dimensional surface meshes. (**C**) Vessel centrelines. (**D**) Automatically identified anatomical landmarks.

The AAo and DAo aortic labels were combined to generate a single aortic mask, while the PA label was retained separately. Surface meshes were generated using the marching-cubes algorithm implemented within the Vascular Modelling Toolkit (VMTK) [16,17]. Vessel contours were also extracted to enable centreline extraction and anatomical landmark detection (**Figure 3**). Centrelines of the aorta and PA were then extracted using automatically generated inlet and outlet seed points derived from the segmented vessel geometry. For the aorta, seed points were generated from the inferior AAo and DAo contours. For the PA, candidate seed points corresponding to the main PA inlet and the right and left PA outlets were identified from the vessel contours, with spatial constraints applied to ensure anatomically plausible seed placement.

Cross-sectional diameters were subsequently calculated along each vessel centreline. For the PA, the bifurcation region was identified automatically based on the divergence of the right and left PA centrelines. Diameters of the main, right and left PA were obtained by averaging cross-sectional measurements within a 5 mm local neighbourhood around each anatomical landmark after excluding outlying values. The PA bifurcation landmark was defined as the point at which the main PA centreline divided into the right and left PA centrelines.

**Figure 4:**
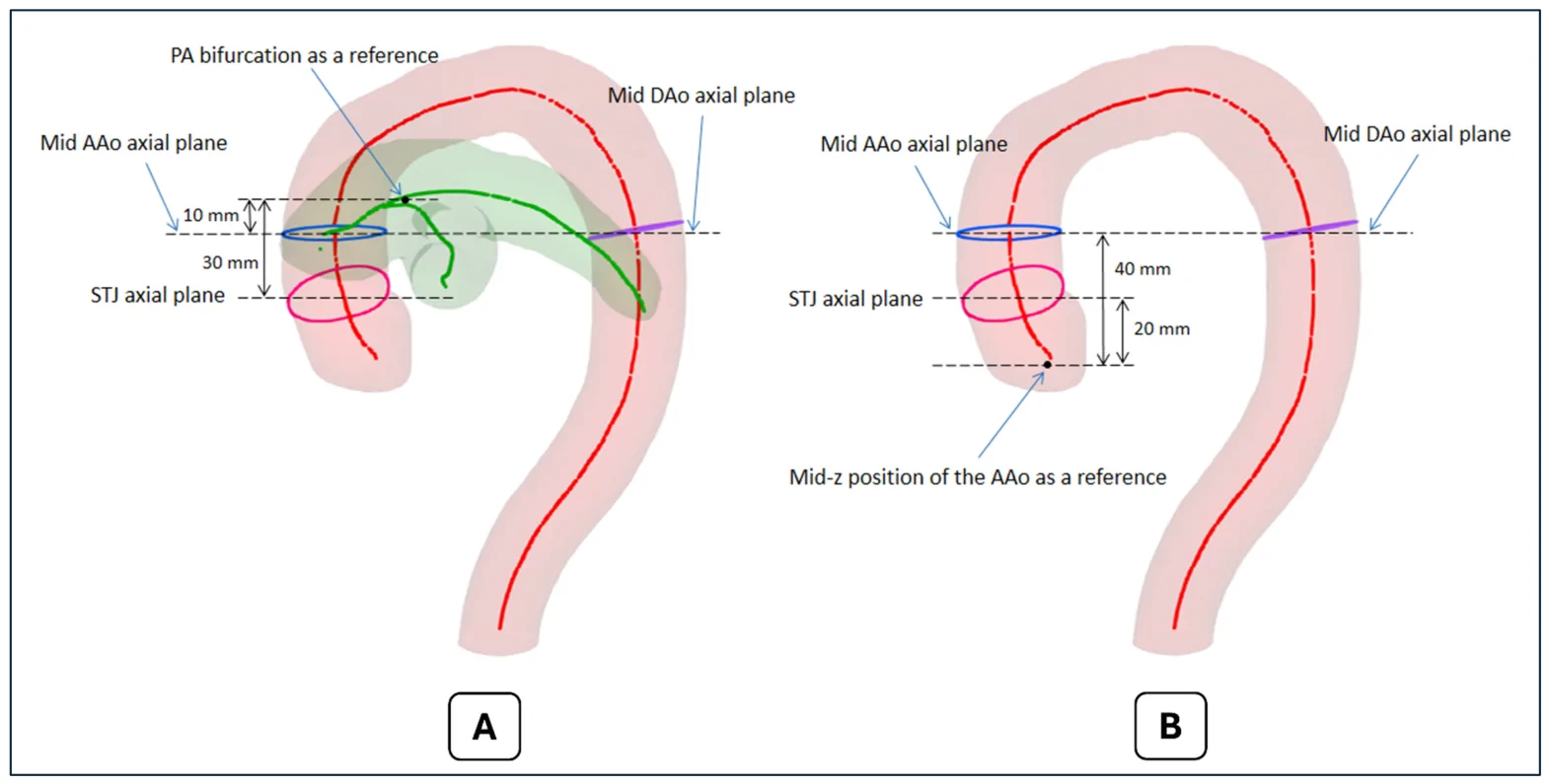
Schematic illustration of the two aortic landmarking strategies: (**A**) primary strategy using the pulmonary artery (PA) bifurcation as the anatomical reference; (**B**) secondary strategy based solely on ascending aorta (AAo) geometry. STJ, sinotubular junction.

For the aorta, measurement landmarks were referenced to the PA bifurcation plane where this could be reliably identified. When the bifurcation plane was not available, the midpoint of the AAo along the z-axis was used as an alternative reference (**Figure 4**). The AAo diameter was calculated by averaging local cross-sectional measurements after removing outliers. The PA-to-aorta (PA/Ao) diameter ratio was subsequently calculated by dividing the main PA diameter by the AAo diameter.

#### 2.4.4. Automated Measurement Failure Assessment 

Acquisitions were classified as failures if the automated pipeline could not successfully complete the processing steps required to generate the intended measurements. Volumetric measurements were derived directly from the segmentation masks and were therefore only affected by segmentation failures. In contrast, automated diameter measurements additionally depended on surface mesh generation, centreline extraction, anatomical landmark identification, and geometric post-processing. Diameter failures were recorded using structured stage-specific logging and categorised according to pipeline stage and failure mode. As diameter measurements were derived from the extracted centrelines, centreline placement was visually reviewed, and cases with incorrect placement were classified as failures. Successful and failed cases were also compared descriptively with respect to voxel spacing, vessel coverage, and segmentation connectivity.

### 2.5. Manual Diameter Measurements

Manual PA and AAo diameter measurements were performed using RadiAnt DICOM Viewer (version 2025.2; Medixant, Poznań, Poland) in a subset of 40 patients with paired WB and BB acquisitions. Manual measurements obtained from paired WB and BB images were used to investigate the relative contributions of image contrast and measurement methodology to the differences observed between automated measurements and routine clinical reports.

### 2.6. Statistical Analysis 

Statistical analyses were performed using IBM SPSS Statistics (version 29.0.1.1, IBM Corp., Armonk, NY, USA). Figures were generated using GraphPad Prism (version 11.0.0, GraphPad Software, San Diego, CA, USA). Continuous measurements were assessed for normality using the Shapiro–Wilk test and reported as mean ± standard deviation when normally distributed or as median (interquartile range) otherwise.

Agreement between automated and reference standard volumetric measurements was assessed using intraclass correlation coefficients (ICC; two-way mixed-effects model, absolute agreement) and Bland–Altman analysis. Inter-observer repeatability was similarly evaluated using ICC between the primary and second-observer volumetric measurements, with agreement between automated and each observer also assessed.

For comparison with qualitative clinical reporting, automated slow-flow volume was classified as present or absent using an exploratory data-derived threshold. Candidate thresholds ranging from 2 to 12 mL in 2 mL increments were evaluated in the model-development cohort, and the threshold associated with the highest Cohen’s kappa was selected. The selected threshold was then applied unchanged to the large clinical application cohort. Agreement between automated slow-flow classification and qualitative clinical reporting was assessed using overall percentage agreement and Cohen’s kappa in patients with documented slow-flow status (*n* = 1350). Automated slow-flow volumes were also compared across qualitative slow-flow severity groups. Additional exploratory analyses assessed agreement with qualitative clinical reporting for slow-flow volume indexed to body surface area (BSA) and expressed as a fraction of total PA volume. Thresholds were selected in the model-development cohort using Cohen’s kappa and applied unchanged to the clinical application cohort.

As an exploratory analysis, automated diameter measurements were compared with routinely reported clinical measurements in patients with successful automated measurements and corresponding clinical report data available (*n* = 972) using (ICC; two-way mixed-effects model, absolute agreement). Failure rates were calculated as the proportion of acquisitions in which automated segmentation or diameter extraction failed and were summarised according to acquisition type and scanner vendor.

## 3. Results

A total of 205 CMR acquisitions were included for model development and independent testing, comprising 106 WB and 99 BB CMR acquisitions. Following an 80/20 split, 84 WB and 79 BB acquisitions were used for model development, while 22 WB and 20 BB acquisitions formed the independent test datasets. Demographic and acquisition characteristics of the development/test dataset and clinical application cohort are summarised in **Table 1**.

### 3.1. Model Performance Evaluation

The automated pipeline demonstrated excellent volumetric agreement and high segmentation accuracy against the manual reference standard (**Table 2**). PA volumetric measurements showed excellent agreement for both the WB and BB models (ICC 0.94 and 0.93, respectively). High spatial overlap was also observed for PA segmentation in both the WB and BB models (Dice 0.86 and 0.80, respectively), with low boundary error (HD95 7.2 and 7.7 mm, respectively). The ascending aorta also demonstrated excellent volumetric agreement (ICC 0.95), with high spatial overlap (Dice 0.95) and low boundary error (HD95 4.9 mm). Model-specific performance metrics for all segmented structures are summarised in **Table S1**.

Segmentation of the slow-flow region demonstrated lower spatial overlap than PA segmentation (Dice 0.61), with an HD95 of 22.7 mm. However, volumetric agreement between automated and manual measurements remained excellent (ICC 0.94). Additional localisation analysis showed that 92.5% of predicted slow-flow volume was contained within the manually segmented whole-PA region, while 0.5% was located >10 mm outside the reference PA. Of the false-positive slow-flow volume, 70.5% was located within the manual PA. Restricting predictions to the manual whole-PA region reduced mean HD95 from 22.7 to 20.9 mm (**Table S2; Figure S1**).

**Figure 5:**
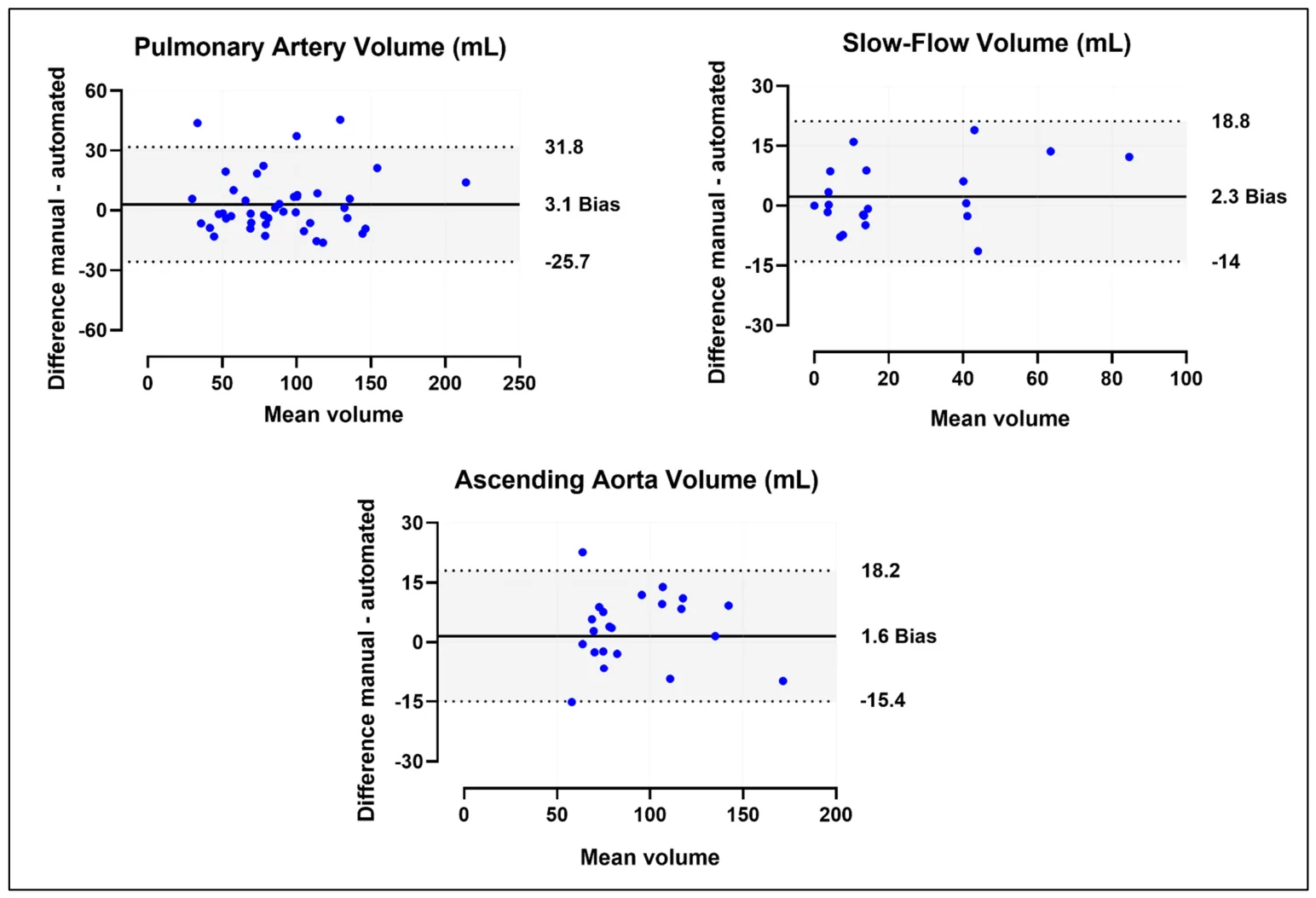
Bland–Altman plots illustrating the agreement and bias between manual and automated volume measurements.

Bland–Altman analysis demonstrated low systematic bias between manual and automated volumetric measurements, with mean biases of 3.1 mL for PA volume, 1.6 mL for ascending aorta volume and 2.3 mL for slow-flow volume (**Figure 5**). Limits of agreement were narrowest for ascending aorta volume and wider for PA and slow-flow volumes.

### 3.2. Inter-Observer Repeatability

Manual volumetric measurements demonstrated good to excellent inter-observer agreement across all structures, with ICCs ranging from 0.86 to 0.97 (**Table S3**). Spatial agreement metrics were similar across all pairwise comparisons (**Table S4**). Dice values ranged from 0.82 to 0.94 for vessel segmentations and from 0.52 to 0.58 for slow-flow segmentations.

### 3.3. Automated Slow-Flow Assessment and Qualitative Clinical Reporting

**Figure 6:**
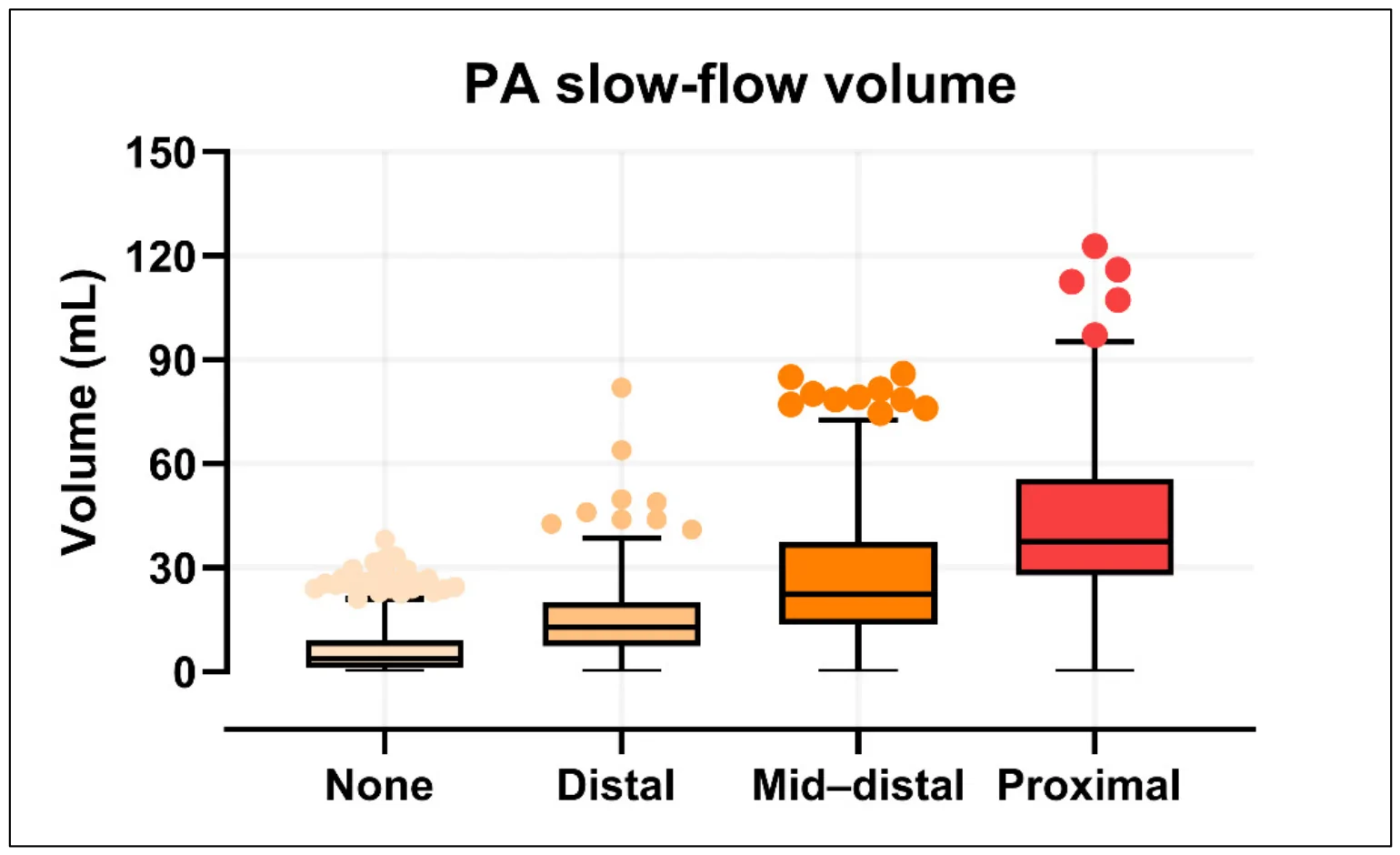
Distribution of automated pulmonary artery slow-flow volume across clinically defined severity groups.

Using the selected 6 mL threshold, automated slow-flow classification demonstrated good agreement with qualitative clinical reporting, with an overall agreement of 83% (95% CI: 81–85%) and a Cohen’s κ of 0.58 (95% CI: 0.53–0.63). The 6 mL threshold achieved a sensitivity of 91%, specificity of 65%, positive predictive value of 86%, and negative predictive value of 75% (**Table 3**). In addition, automated slow-flow volumes increased progressively across clinically defined severity groups, with higher values observed as the extent of slow-flow increased (**Figure 6**). Exploratory analyses demonstrated similar agreement with qualitative clinical reporting for absolute, BSA-indexed and fractional slow-flow volume measurements (**Table S5**).

### 3.4. Automated Diameter Measurements Versus Routine Clinical Reporting 

Automated diameter measurements were lower than routine clinical measurements for both the PA and AAo (**Table 4**). Agreement was moderate for PA diameter (ICC 0.71, 95% CI 0.68–0.74) and AAo diameter (ICC 0.72, 95% CI 0.69–0.75), with automated measurements approximately 4 mm lower than clinically reported values. In contrast, the derived PA/Ao ratio demonstrated good agreement with clinical measurements (ICC 0.89, 95% CI 0.86–0.91), with minimal difference in mean values between methods.

Additional manual measurements performed on paired WB and BB acquisitions demonstrated that PA and ascending aortic diameters measured on BB images were approximately 2 mm larger than those measured on WB images. Similarly, manual measurements obtained from WB images were approximately 2 mm larger than the corresponding automated measurements. In contrast, the PA/Ao ratio remained relatively stable across measurement approaches (**Table S6**).

### 3.5. Failure Rate

Failure rates varied across measurement output and scanner vendors (**Table 5**). Volumetric measurement failures were uncommon across both acquisition types. In the GE cohort, failure rates were 1.3% for WB and 4.0% for BB volumetric measurements, respectively, while no WB volumetric measurement failures were observed in the Siemens cohort.

In contrast, automated diameter extraction failed in 363/1431 GE acquisitions (25.4%) and 8/40 Siemens acquisitions (20.0%). In the GE cohort, the predominant failure modes were failure to identify the left PA outlet (223/363; 61.4%) and PA bifurcation (95/363; 26.2%) (**Table S7**). Successful and failed GE cases showed broadly similar voxel spacing, vessel coverage and segmentation connectivity, with differences observed in some measures of geometric extent. In the smaller Siemens cohort, failed cases showed lower aortic coverage and geometric extent, although this comparison was limited by the small number of failures (**Table S8**).

## 4. Discussion

This study developed and validated a fully automated CMR pipeline for pulmonary artery volumetric and slow-flow quantification from routine WB and BB acquisitions and then demonstrated its feasibility in a large clinical cohort. Automated PA volumetric measurements showed excellent agreement with manual reference standard measurements, with similarly high agreement observed for the ascending aorta. Automated slow-flow volumes demonstrated good agreement with manual reference measurements and were consistent with qualitative clinical reporting, with increasing volumes observed across clinically defined slow-flow severity groups. In contrast, automated diameter measurements were systematically lower than routine clinical measurements, and diameter extraction showed higher failure rates than volume extraction across vendors. These findings indicate that the proposed pipeline performs most robustly for automated volumetric and slow-flow quantification.

The excellent agreement between automated and manual volumetric measurements suggests that the model captured the overall vascular extent reliably, even when local segmentation differences were present. This relationship between segmentation performance and volumetric agreement differed between structures. In the ascending aorta, high Dice scores, low HD95 values and narrow Bland–Altman limits of agreement indicated consistent segmentation and volumetric quantification. This is consistent with previous automated vascular segmentation studies, where larger and more anatomically regular vessels such as the aorta generally demonstrate strong segmentation performance [18,19]. In contrast, the PA showed lower spatial overlap and higher boundary error, likely reflecting its more complex branching anatomy and greater variation in vessel shape compared with the ascending aorta. Importantly, these findings highlight that segmentation performance should be considered in relation to the intended measurement output, particularly when the primary output is volumetric quantification rather than precise boundary localisation. This supports previous recommendations that different segmentation metrics capture different aspects of performance [20,21].

This distinction was most evident for the slow-flow region, where spatial overlap and boundary agreement were lower despite strong volumetric agreement. Volumetric and spatial metrics assess different aspects of performance, and agreement in slow-flow volume does not necessarily imply identical anatomical localisation. The additional containment analysis showed that most predicted slow-flow remained within the manually segmented PA, while restricting predictions to the manual PA resulted in only a small reduction in HD95. These findings suggest that elevated HD95 was not primarily driven by distant extra-PA predictions, but may reflect differences in the spatial distribution and extent of slow-flow within the PA. Unlike fixed anatomical structures, slow-flow represents areas of altered signal that may vary in shape, intensity and extent across slices. Together with the relatively coarse through-plane resolution of BB acquisitions, this may make precise spatial correspondence more challenging. The variability in delineating slow-flow was also reflected in the inter-observer analysis, with higher Dice scores and lower HD95 values for agreement between the automated model and manual annotations than between human observers. Accordingly, volumetric and spatial metrics should be considered complementary when evaluating automated slow-flow quantification.

The slow-flow analysis provides an additional assessment of whether the automated measurements reflect the patterns described in routine clinical reporting. Automated slow-flow volumes showed good agreement with qualitative clinical reporting and increased across clinically defined severity groups. These findings are important because they demonstrate that automated measurements reflect clinically reported slow-flow severity, which is routinely described qualitatively, for example, as proximal or distal involvement. As absolute slow-flow volume may be confounded by differences in body size and PA dilatation, additional analyses indexing slow-flow volume to BSA or total PA volume were performed. Absolute, BSA-indexed and fractional slow-flow volume measurements demonstrated similar agreement with qualitative clinical reporting. Previous work has used semi-quantitative visual scoring to provide a more structured assessment of pulmonary arterial slow-flow, although this remains subjective [6]. The automated approach used in this study extends this concept by providing a continuous volumetric measure of slow-flow signal, allowing more objective and standardised quantification of slow-flow extent. However, the 6 mL threshold remains an exploratory classification threshold rather than an established physiological cut-off.

The weaker agreement observed for automated diameter measurements likely reflects both methodological and acquisition-related differences. PA diameter is an established quantitative CMR measurement and may be assessed using different image contrasts, including BB and WB techniques [22]. In this study, routine clinical diameters were measured on BB images, whereas automated diameters were derived from WB segmentation masks. In addition, automated diameter extraction was based on centreline-derived cross-sections and local averaging, whereas routine clinical measurements are typically obtained from reader-selected planes. Additional manual measurements suggested that the observed differences between the automated and routine clinical diameter measurements were influenced by both image contrast and measurement methodology. Despite systematic differences in absolute PA and ascending aortic diameters, the PA/Ao ratio remained largely preserved and demonstrated good agreement with routine clinical reporting, suggesting that these systematic differences influenced both vessels similarly rather than indicating poor segmentation. Diameter extraction is also more sensitive than volume extraction to landmark placement, bifurcation anatomy and local vessel geometry [4]. Therefore, automated diameter measurements should be interpreted as exploratory outputs derived through geometric post-processing, whereas volumetric measurements represent the primary validated outputs of the framework.

The failure rate analysis further highlights the difference between segmentation-derived volumetric measurements and post-processing-derived diameter measurements. Volume extraction showed low failure rates, whereas diameter extraction was more sensitive to downstream branch and landmark identification. Most diameter-extraction failures were associated with difficulties identifying the left PA outlet and PA bifurcation. The relatively coarse through-plane resolution of routine WB CMR, with approximately 10 mm slice spacing, may contribute to these failures by producing a sparsely sampled and highly anisotropic three-dimensional representation of vascular anatomy. These findings suggest that future optimisation should focus not only on segmentation accuracy but also on the robustness of downstream measurement pipelines before automated diameter measurements can be considered suitable for routine clinical use.

Several limitations of this study should be acknowledged. First, the independent test sets were relatively small, comprising 22 WB and 20 BB acquisitions, which may limit the precision of the reported performance estimates. In addition, this was a single-centre study, and although the model included data from both GE and Siemens scanners, Siemens representation was limited to WB acquisitions. Siemens BB acquisitions were therefore not included in the BB volume analysis, and the generalisability of the BB model across scanner vendors remains to be established. This is particularly relevant for slow-flow quantification, as the appearance of slow-flow on BB CMR may be influenced by sequence design and acquisition parameters. Further validation across institutions, scanner vendors and BB acquisition protocols is therefore required to establish the robustness and generalisability of the proposed framework [23]. Second, although automated slow-flow segmentation was technically validated against manual reference measurements, agreement in the large clinical cohort was assessed against routine qualitative clinical reporting. Therefore, some apparent disagreements may reflect variability in clinical interpretation rather than errors in the automated method. Third, automated diameter measurements were compared with routine clinical diameter measurements, which may be influenced by reader-selected measurement planes and may therefore explain the weaker agreement observed. Furthermore, automated diameters were derived from WB segmentation masks using a centreline-based approach, whereas routine clinical measurements were performed on BB images. Therefore, the observed bias may reflect differences in image contrast and measurement strategy, rather than limitations of the automated pipeline alone. Finally, automated diameter measurements were derived from segmentation masks using geometric post-processing rather than directly predicted by the model. Clinical agreement analyses were restricted to cases with successful automated diameter extraction and a corresponding clinical measurement from the same MRI examination. This may have introduced selection bias, potentially overestimating agreement in an unselected clinical population. The remaining diameter-extraction failures therefore represent an important limitation that requires further optimisation before routine clinical application.

The present study focused on the technical validation of automated CMR-derived PA volumetric and slow-flow measurements. Their clinical utility in PH diagnosis and haemodynamic phenotyping will be evaluated in a subsequent clinical study.

## 5. Conclusions

This study demonstrates the feasibility of a fully automated CMR pipeline for pulmonary artery volumetric and slow-flow quantification from routine WB and BB acquisitions. Automated volumetric measurements showed excellent agreement with manual reference measurements, supporting volumetric quantification as a robust output of the pipeline. Automated slow-flow volume also demonstrated good agreement with manual reference measurements and provided a continuous quantitative measure of slow-flow regions. Although automated diameter measurements were explored, further optimisation of the downstream measurement pipeline is required before they can be considered for routine clinical application. Overall, these findings demonstrate the potential of automated CMR-derived volumetric and slow-flow measurements to support more efficient and standardised pulmonary artery assessment, while further validation across scanner vendors, institutions and acquisition protocols, particularly for slow-flow quantification, is needed to establish the generalisability of the proposed framework.

## Figures and Tables

**Table 1:** Demographic and acquisition characteristics of the model development/test dataset and clinical application cohort.

Characteristic	Development/Test Dataset	Clinical Application Cohort
	**Patient characteristics**	
**Patients, n**	118	1471
**Age, years**	64 ± 14	64 ± 13
**Sex, male/female**	56/62	530/941
**PH diagnosis, PH/non-PH**	86/32	1284/187
	**Acquisition characteristics**	
**Total CMR acquisitions, n**	205	2902
**GE white-blood acquisitions, n**	87	1431
**Siemens white-blood acquisitions, n**	19	40
**GE black-blood acquisitions, n**	99	1431

**PH** = pulmonary hypertension; **CMR** = cardiovascular magnetic resonance. Values are presented as mean ± SD or n, as appropriate.

**Table 2:** Technical performance of the automated segmentation pipeline against manual reference standard measurements.

Structure	ICC (95% CI)	Dice (95% CI)	HD95 (95% CI)
**Ascending Aorta (WB)**	0.95 (0.89–0.98)	0.95 (0.93–0.96)	4.9 (0.3–9.5)
**Pulmonary artery (WB)**	0.94 (0.86–0.97)	0.86 (0.85–0.88)	7.2 (4.8–9.6)
**Pulmonary artery (BB)**	0.93 (0.82–0.97)	0.80 (0.74–0.86)	7.7 (4.1–11.3)
**Slow-flow (BB)**	0.94 (0.85–0.97)	0.61 (0.51–0.70)	22.7 (13.6–31.8)

**ICC** = Intraclass Correlation Coefficient (95% CI); **Dice** = Dice similarity coefficient; **HD95** = 95^th^ percentile Hausdorff Distance (mm).

**Table 3:** Agreement between automated slow-flow classification and qualitative clinical reporting (*n* = 1350).

Clinical Report	Automated Absent (<6 mL)	Automated Present (≥6 mL)	Total
**No slow-flow**	**255 (65%)**	139 (35%)	394
**Slow-flow present**	85 (9%)	**871 (91%)**	956
**Total**	340	1010	1350

Values are presented as number (%). Percentages are calculated within each clinical reporting category.

**Table 4:** Comparison of automated diameter measurements with routine clinical reporting for the pulmonary artery and ascending aorta (*n* = 972).

Measurement	Clinical Report	Automated	ICC (95% CI)
**Pulmonary artery diameter (mm)**	31.5 ± 5.7	27.5 ± 5.0	0.71 (0.68–0.74)
**Ascending aorta diameter (mm)**	31.4 ± 4.0	27.6 ± 3.8	0.72 (0.69–0.75)
**PA/Ao ratio**	1.02 ± 0.19	1.01 ± 0.19	0.89 (0.86–0.91)

**ICC** = intraclass correlation coefficient; **CI** = confidence interval; **PA** = pulmonary artery; **Ao** = aorta. Values are presented as mean ± SD.

**Table 5:** Failure rates of automated CMR measurements by vendor and acquisition type.

CMR Metric	Vendor	Acquisitions, n	Failure Rate, n (%)
**White-blood volume**	GE	1431	18 (1.3)
**White-blood diameter**		1431	363 (25.4)
**Black-blood volume**		1431	57 (4.0)
**White-blood volume**	Siemens	40	0
**White-blood diameter**		40	8 (20.0)

Values are presented as n (%).

## Data Availability

De-identified data within the ASPIRE registry can be made available for analysis to researchers who provide a methodologically sound proposal that falls within the aims of the ASPIRE registry. The ASPIRE Data Management Committee will assess each proposal and make a decision within three months of submission. A data access agreement signed by the lead researcher and a data sharing agreement between Sheffield Teaching Hospitals NHS Foundation Trust and the recipient institution will be required before data are shared. Information and application forms are available at **ASPIRE Registry** (https://bit.ly/aspire-registry, accessed on 11 August 2026). The trained models can be made available upon reasonable request for research purposes by non-commercial entities. Requests should be directed to the corresponding author.

## Supplementary Materials

The following supporting information can be downloaded at: https://www.mdpi.com/article/10.3390/diagnostics16193257/s1, Table S1: Technical validation metrics for all structures segmented by the white-blood and black-blood CMR models; Table S2: Additional anatomical localisation analysis of automated slow-flow segmentation in the independent black-blood test set; Table S3: Inter-observer repeatability of manual segmentation volumes between the reference annotations and a second observer; Table S4: Performance of the automated framework in the inter-observer repeatability subset (*n* = 30 acquisitions); Table S5: Exploratory comparison of agreement between absolute, BSA-indexed and fractional pulmonary artery slow-flow volume measurements and qualitative clinical reporting; Table S6: Mean differences between manual, automated and clinically reported pulmonary artery and aortic diameter measurements in the 40-patient paired WB/BB subset; Table S7: Detailed diameter-extraction failure modes by vendor; Table S8: Descriptive acquisition and segmentation-geometry characteristics of successful and failed diameter extraction; Figure S1: Representative examples of automated and manual slow-flow localisation in the independent BB test set.
