# Supplementary material for: Automated CMR Quantification of Pulmonary Artery Size-Based and Slow-Flow Metrics in Pulmonary Hypertension
Source: Diagnostics (Basel). 2026 Oct 8;16(19):3257. doi: 10.3390/diagnostics16193257 (PMC13664574; doi:10.3390/diagnostics16193257)
Supplement: Supplementary file 1 [file diagnostics-16-03257-s001.zip › Supplementary File S1. CLAIM Checklist.pdf]

Checklist for Artificial Intelligence in Medical Imaging (CLAIM): 2024 Update

Section / Topic	No.	Item	Page / Line	No	NA
TITLE / ABSTRACT					
	1	Identification as a study of AI methodology, specifying the category of technology used (e.g., deep learning)	2/5		
ABSTRACT					
	2	Summary of study design, methods, results, and conclusions	2		
INTRODUCTION					
	3	Scientific and/or clinical background, including the intended use and role of the AI approach	4 – 5		
	4	Study aims, objectives, and hypotheses	5		
METHODS					
Study Design	5	Prospective or retrospective study	5/9		
	6	Study goal	5		
Data	7	Data sources	5		
	8	Inclusion and exclusion criteria	5		
	9	Data pre-processing	7 – 8		
	10	Selection of data subsets	5		
	11	De-identification methods	5/19 – 20		
	12	How missing data were handled	5/20 – 21		
	13	Image acquisition protocol	5 – 6		
Reference Standard	14	Definition of method(s) used to obtain reference standard	6		
	15	Rationale for choosing the reference standard	6		
	16	Source of reference standard annotations	6		
	17	Annotation of test set	6		
	18	Measures of inter- and intra-rater variability of features described by the annotators	6		
Data Partitions	19	How data were assigned to partitions	7		
	20	Level at which partitions are disjoint	7		
Testing Data	21	Intended sample size		✓	

Section / Topic	No.	Item	Page / Line	No	NA
Model	**22**	Detailed description of model	**7 – 9**		
	**23**	Software libraries, frameworks, and packages	**7 – 9**		
	**24**	Initialization of model parameters	**8/2 – 3**		
Training	**25**	Details of training approach	**7 – 8**		
	**26**	Method of selecting the final model	**8 / 8 – 10**		
	**27**	Ensembling techniques	**8 / 8 – 10**		
Evaluation	**28**	Metrics of model performance	**8 / 18 – 21**		
	**29**	Statistical measures of significance and uncertainty	**11**		
	**30**	Robustness or sensitivity analysis	**11**		
	**31**	Methods for explainability or interpretability			✓
	**32**	Evaluation on internal data	**7 / 3 – 5**		
	**33**	Testing on external data		✓	
	**34**	Clinical trial registration			✓
**RESULTS**					
Data	**35**	Numbers of patients or examinations included and excluded	**12 / 2 – 5**		
	**36**	Demographic and clinical characteristics of cases in each partition	**20**		
Model performance	**37**	Performance metrics and measures of statistical uncertainty	**12 + 21**		
	**38**	Estimates of diagnostic performance and their precision	**12**		
	**39**	Failure analysis of incorrect results	**14 + 22**		
**DISCUSSION**					
	**40**	Study limitations	**17 – 18**		
	**41**	Implications for practice, including intended use and/or clinical role	**16 / 24 – 26**		
**OTHER INFORMATION**					
	**42**	Provide a reference to the full study protocol or to additional technical details	**5 – 11**		
	**43**	Statement about the availability of software, trained model, and/or data	**29**		
	**44**	Sources of funding and other support; role of funders	**28**		

* Indicate page and/or line number for each checklist item that is present. NA = not applicable.

Tejani AS, Klontzas ME, Gatti AA, et al. Checklist for Artificial Intelligence in Medical Imaging (CLAIM):2024 Update. Radiol Artif Intell 2024;6(4):e240300. https://doi.org/10.1148/ryai.240300
