# Supplementary material for: Automated CMR Quantification of Pulmonary Artery Size-Based and Slow-Flow Metrics in Pulmonary Hypertension
Source: Diagnostics (Basel). 2026 Oct 8;16(19):3257. doi: 10.3390/diagnostics16193257 (PMC13664574; doi:10.3390/diagnostics16193257)
Supplement: Supplementary file 1 [file diagnostics-16-03257-s001.zip › Supplementary materials.pdf]

Supplementary materials

Table S1. Technical validation metrics for all structures segmented by the white-blood and black-blood CMR models.

Structure	Manual volume	Automated volume	ICC (95% CI)	Dice (95% CI)	HD95 (95% CI)
**White-blood model (n = 22)**					
**Pulmonary Artery**	92.9 (75.5 –110.3)	90.0 (73.2 – 106.9)	0.94 (0.86 – 0.97)	0.86 (0.85 – 0.88)	7.2 (4.8 – 9.6)
**Ascending Aorta**	94.1 (80.7 –107.5)	91.8 (78.4 – 104.2)	0.95 (0.89 – 0.98)	0.95 (0.93 – 0.96)	4.9 (0.3 – 9.5)
**Descending Aorta**	76.2 (69.1 –83.3)	75.2 (69.2 –81.1)	0.94 (0.87 – 0.98)	0.94 (0.93 – 0.95)	2.3 (0.6 – 4.1)
**Black-blood model (n = 20)**					
**Pulmonary Artery**	88.9 (69.5 –108.3)	85.6 (66.6 – 104.7)	0.93 (0.82 – 0.97)	0.80 (0.74 – 0.86)	7.7 (4.1 – 11.3)
**Ascending Aorta**	76.4 (67.5 –85.3)	76.7 (68.3 –85.0)	0.91 (0.79 –0.97)	0.94 (0.93 – 0.96)	5.2 (1.1 – 9.3)
**Descending Aorta**	30.1 (25.1 –35.1)	28.8 (24.4 –33.2)	0.93 (0.83 –0.97)	0.93 (0.91 – 0.95)	2.7 (1.1 – 4.2)
**Slow-flow**	25.4 (13.1 – 37.7)	23.3 (12.7 –33.9)	0.94 (0.85 – 0.97)	0.61 (0.51 – 0.70)	22.7 (13.6 – 31.8)

ICC = intraclass correlation coefficient; **Dice** = Dice similarity coefficient; **HD95** = 95th percentile Hausdorff distance. Values are presented as mean (95% confidence interval). Manual and automated volumes are reported in millilitres (mL) and HD95 in millimetres (mm).

Table S2. Additional anatomical localisation analysis of automated slow-flow segmentation in the independent black-blood test set.

Analysis	Metric	Result	Denominator / interpretation
**Cohort**	Independent BB test acquisitions	20	All independent BB test cases
	Cases with slow-flow present in both reference and prediction	18	Cases contributing to HD95
**Anatomical containment**	Predicted slow-flow within manual whole PA	92.5%	Pooled predicted slow-flow volume
	Predicted slow-flow outside manual whole PA	7.5%	Pooled predicted slow-flow volume
	Outside-PA slow-flow per case	5.7% (IQR 2.0–13.5%)	18 cases with predicted slow-flow present
	Predicted slow-flow >5 mm outside manual PA	3.7%	Pooled predicted slow-flow volume
	Predicted slow-flow >10 mm outside manual PA	0.5%	Pooled predicted slow-flow volume
**False-positive localisation**	Slow-flow false-positive volume within manual PA	70.5%	Pooled slow-flow false-positive volume
	Slow-flow false-positive volume outside manual PA	29.5%	Pooled slow-flow false-positive volume
**Effect on HD95**	Original slow-flow HD95	22.7 mm	Mean across 18 cases with both masks present
	HD95 after restricting prediction to manual whole PA	20.9 mm	Diagnostic analysis only; uses manual PA information
	Mean HD95 reduction after restriction	1.8 mm	Diagnostic analysis only
	Median HD95 reduction after restriction	0.0 mm	Diagnostic analysis only

BB = black-blood; **PA** = pulmonary artery; **HD95** = 95th percentile Hausdorff distance. Whole PA was reconstructed as the union of the final PA and slow-flow labels. Pooled percentages are volume-weighted across all predictions. The diagnostic HD95 analysis used the manual whole-PA mask and was performed only to investigate the contribution of extra-PA predictions to surface-distance error; it does not represent deployable model performance.

Table S3. Inter-observer repeatability of manual segmentation volumes between the reference annotations and a second observer.

Acquisition	Structure	ICC (95% CI)
**White-blood (n = 15)**	Pulmonary artery	0.89 (0.47 – 0.97)
	Ascending aorta	0.93 (0.80 – 0.98)
	Descending aorta	0.93 (0.60 – 0.98)
**Black-blood (n = 15)**	Pulmonary artery	0.97 (0.91 – 0.99)
	Ascending aorta	0.86 (0.34 – 0.96)
	Descending aorta	0.92 (0.76 – 0.97)
	Slow-flow volume	0.95 (0.86 – 0.98)

ICC = intraclass correlation coefficient; **CI** = confidence interval.

Table S4. Performance of the automated framework in the inter-observer repeatability subset (n = 30 acquisitions)**.**

Acquisition	Structure	Reference vs Observer 2		Reference vs AI		Observer 2 vs AI	
		Dice	HD95 (mm)	Dice	HD95 (mm)	Dice	HD95 (mm)
**WB**	PA	0.84	5.10	0.88	1.15	0.85	4.15
	AAo	0.89	1.39	0.94	0.97	0.90	1.20
	DAo	0.90	1.69	0.94	0.98	0.91	1.71
**BB**	PA	0.82	7.05	0.83	1.15	0.83	6.76
	AAo	0.92	9.90	0.94	0.81	0.92	8.97
	DAo	0.91	4.62	0.93	0.68	0.90	5.67
	Slow-flow	0.52	33.22	0.58	7.58	0.55	18.91

Reference = manual reference annotation; **AI** = automated segmentation; **WB** = white-blood; **BB** = black-blood; **PA** = pulmonary artery; **AAo** = ascending aorta; **DAo** = descending aorta. Values are presented as mean Dice similarity coefficient and mean 95th percentile Hausdorff distance (HD95).

Table S5. Exploratory comparison of agreement between absolute, BSA-indexed and fractional pulmonary artery slow-flow volume measurements and qualitative clinical reporting.

Measurement	Agreement	Cohen's κ
**Absolute slow-flow volume ≥ 6 mL**	83.4%	0.58
**BSA-indexed slow-flow volume ≥ 3 mL/m²**	83.8%	0.58
**Slow-flow fraction ≥ 10%**	81.5%	0.56

Table S6. Mean differences between manual, automated and clinically reported pulmonary artery and aortic diameter measurements in the 40-patient paired WB/BB subset.

Measurement	BB Manual	WB Manual	WB Automated	Mean Difference (BB Manual – WB Manual)	Mean Difference (WB Manual – WB Automated)
**PA diameter (mm)**	30.7 ± 4.4	28.7 ± 4.9	26.2 ± 4.4	1.95	2.55
**AAo diameter (mm)**	31.4 ± 3.5	29.7 ± 3.7	27.6 ± 3.9	1.73	2.06
**PA/Ao ratio**	0.98 ± 0.14	0.97 ± 0.14	0.95 ± 0.15	0.01	0.02
**WB** = white-blood; **BB** = black-blood; **PA** = pulmonary artery; **AAo** = ascending aorta. Values are presented as mean ± SD.					

Table S7. Detailed diameter extraction failure modes by vendor.

Vendor	Cohort, n	Failures, n (%)	Failure mode	n	% of failures	% of cohort
**GE**	1,431	363 (25.4)	Left PA outlet not identified	223	61.4	15.6
			PA bifurcation not identified	95	26.2	6.6
			PA-trunk diameter window empty	20	5.5	1.4
			Aortic branch split invalid	10	2.8	0.7
			Right PA outlet not identified	9	2.5	0.6
			Descending-aorta landmark split invalid	4	1.1	0.3
			Centreline segment too short after resampling	1	0.3	0.1
			Zero-length centreline segment	1	0.3	0.1
**Siemens**	40	8 (20.0)	Left PA outlet not identified	4	50.0	10.0
			PA-trunk diameter window empty	2	25.0	5.0
			PA bifurcation not identified	1	12.5	2.5
			Descending-aorta landmark split invalid	1	12.5	2.5

Table S8. Descriptive acquisition and segmentation-geometry characteristics of successful and failed diameter extraction.

Vendor	Characteristic	Successful, median (IQR)	Failed, median (IQR)
GE	Image z-slices	30 (28-30)	30 (28-30)
	In-plane spacing, mm	0.94 (0.94-0.94)	0.94 (0.94-0.94)
	Through-plane spacing, mm	10.0 (10.0-10.0)	10.0 (10.0-10.0)
	Ascending-aorta slice coverage	11 (10-12)	11 (10-12)
	PA slice coverage	5 (5-6)	6 (5-6)
	Descending-aorta slice coverage	19 (18-20)	19 (17-20)
	PA lateral extent, mm	114.4 (105.0-123.8)	111.6 (100.3-120.9)
	Ascending-aorta through-plane extent, mm	110 (100-120)	110 (100-120)
	Descending-aorta through-plane extent, mm	190 (180-200)	180 (170-200)
Siemens	Image z-slices	25 (25-25)	25 (25-25)
	In-plane spacing, mm	1.33 (1.33-1.33)	1.33 (1.33-1.33)
	Through-plane spacing, mm	10.0 (10.0-10.0)	10.0 (10.0-10.0)
	Ascending-aorta slice coverage	11 (9.8-11.3)	9 (8-10)
	PA slice coverage	5 (5-6)	5 (5-5)
	Descending-aorta slice coverage	18 (17.8-19)	17.5 (15.5-18.3)
	PA lateral extent, mm	114.2 (105.9-123.8)	101.0 (95.0-114.9)
	Ascending-aorta through-plane extent, mm	110 (97.5-112.5)	90 (80-100)
	Descending-aorta through-plane extent, mm	180 (177.5-190)	175 (155-182.5)

Note: All PA, ascending-aortic and descending-aortic masks in both successful and failed groups comprised a single connected component. Values are median (IQR). IQR = interquartile range; PA = pulmonary artery.

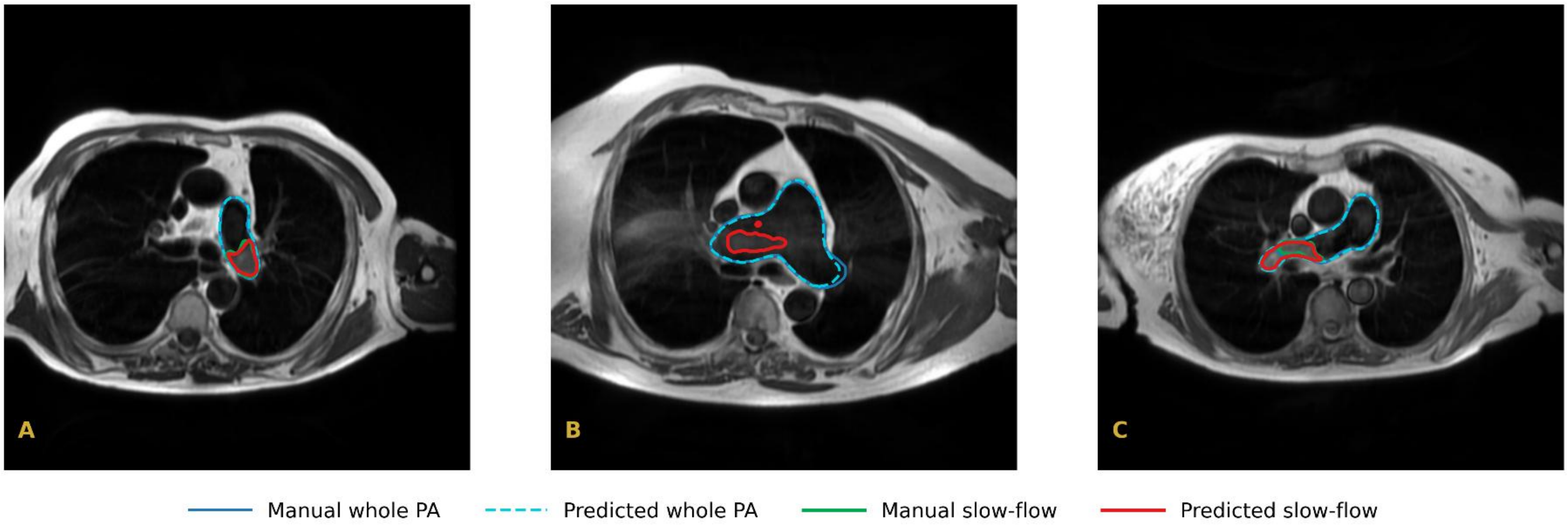

Figure S1. Representative examples of automated slow-flow localisation in the independent black-blood test set. Manual and predicted whole-PA boundaries and manual and predicted slow-flow segmentations are overlaid on representative axial BB CMR images. (**A**) Good spatial agreement. (**B**) High HD95 despite complete containment of predicted slow-flow within the manual whole-PA region, illustrating within-PA and adjacent-slice disagreement. (**C**) Predicted slow-flow extending outside the manual whole-PA boundary while remaining within the predicted whole-PA region.
